# Gelatin nanoparticles enhance delivery of hepatitis C virus recombinant *NS2* gene

**DOI:** 10.1371/journal.pone.0181723

**Published:** 2017-07-26

**Authors:** Salwa Sabet, Marina A. George, Haidan M. El-Shorbagy, Heba Bassiony, Khaled Y. Farroh, Tareq Youssef, Taher A. Salaheldin

**Affiliations:** 1 Department of Zoology, Faculty of Science, Cairo University, Giza, Egypt; 2 Department of Photochemistry and Photobiology, National Institute of Laser Enhanced Sciences, Cairo University, Giza, Egypt; 3 Nanotechnology and Advanced Materials Central Lab., Agriculture Research Center, Giza, Egypt; 4 Mostafa El-Sayed Nanotechnology Research Center, British University in Egypt, El Sherouk, Egypt; Advanced Centre for Treatment Research and Education in Cancer, INDIA

## Abstract

**Background:**

Development of an effective non-viral vaccine against hepatitis C virus infection is of a great importance. Gelatin nanoparticles (Gel.NPs) have an attention and promising approach as a viable carrier for delivery of vaccine, gene, drug and other biomolecules in the body.

**Aim of work:**

The present study aimed to develop stable Gel.NPs conjugated with nonstructural protein 2 (*NS2*) gene of Hepatitis C Virus genotype 4a (HCV4a) as a safe and an efficient vaccine delivery system.

**Methods and results:**

Gel.NPs were synthesized and characterized (size: 150±2 nm and zeta potential +17.6 mv). *NS2* gene was successfully cloned and expressed into E. coli M15 using pQE-30 vector. Antigenicity of the recombinant *NS2* protein was confirmed by Western blotting to verify the efficiency of *NS2* as a possible vaccine. Then *NS2* gene was conjugated to gelatin nanoparticles and a successful conjugation was confirmed by labeling and imaging using Confocal Laser Scanning Microscope (CLSM). Interestingly, the transformation of the conjugated *NS2*/Gel.NPs complex into E. coli DH5-α was 50% more efficient than transformation with the gene alone. In addition, conjugated *NS2*/Gel.NPs with ratio 1:100 (w/w) showed higher transformation efficiency into E. coli DH5-α than the other ratios (1:50 and 2:50).

**Conclusion:**

Gel.NPs effectively enhanced the gene delivery in bacterial cells without affecting the structure of *NS2* gene and could be used as a safe, easy, rapid, cost-effective and non-viral vaccine delivery system for HCV.

## Introduction

Egypt has the highest prevalence of Hepatitis C Virus (HCV) infection in the world, making it one of the major public health challenges facing the country. Approximately 14.7% (about 10 million) of the Egyptian population are anti-HCV positive, mainly genotype 4a [1,2]. Only 20–30% of HCV patients are able to clear the virus spontaneously [3], while the remaining 70–80% develop chronic infection and have a risk to develop cirrhosis and hepatocellular carcinoma (HCC) [4,5].

Approved combination therapy of Poly Ethylene Glycated (PEGylated) Interferon and Ribavirin is expensive and causes many side effects including anemia and decrease of the neutrophils count [6,7]. Moreover, only about 55% of patients respond to the treatment and can successfully clear the virus depending on virological, immunological and genetic factors [8,9]. New regimens of Sofosbuvir provide HCV–g4 high cure rates by inhibiting HCV polymerase but are still the highest cost treatment [10]. As prevention is better than treatment, and to avoid the expected mortality from HCV-related cirrhosis or HCC in the next decade; an effective vaccine against HCV should be developed [11]. For this purpose; nonstructural (NS2) protease domain, the most genetically conserved viral antigen among HCV genotypes, has been used for the induction of cellular immunity in animal models in different vaccine studies [12], this might help in the design of a vaccine against the predominant genotype 4a.

Non-structural protein 2 (NS2) is an integral membrane protein (23-kDa of 217 amino acids) [13]. The N-terminus of NS2 (residues 1–94) forms three transmembrane domains, while the C-terminus (residues 94–217) exists in the cytoplasm [14]. NS2 is suggested to play a vital role in HCV assembly and replication through its interaction with structural (E1 and E2) and non-structural (p7, NS3-4A, and NS5A) proteins [15,16]. NS2 is an essential key enzyme for the viral life cycle, making it an excellent candidate molecule for the development of antiviral therapies and vaccines against HCV infection [17]. The safest and most efficient method of gene delivery into the human cell is still a point of interest in biotechnological research. Direct administration of naked DNA is the easiest route but limited only to rigid tumors [18] and muscle tissue [19]. In addition, naked DNA can be rapidly degraded by blood enzymes and repulsed with the negatively charged cell membrane that consequently inhibits efficient delivery [20]. Viral carriers are prohibited by Food and Drug Administration (FDA) because of their high risk of toxicity, inflammatory and immune responses [21,22]. Recently, biocompatible nanomaterials are promising carriers with low toxicity and well-controlled gene delivery [23]. Gelatin nanoparticles (Gel.NPs) have significant uses in biomedical and pharmaceutical research. Gelatin is nontoxic, biodegradable, bioactive, inexpensive, non-immunogenic and biocompatible to human tissues. Gel.NPs are very efficient in vaccine, gene or drug delivery in the body [24]. Properties of Gel.NPs such as size, swelling behavior, and thermal properties; depend on the crosslinking degree between cationic and anionic groups that could be controlled during preparation. Gel.NPs can be prepared by the desolvation/coacervation or the emulsion methods [24,25].

The present study aimed to investigate NS2 antigenicity in order to develop a safe, rapid, easy, non-immunogenic, more effective and efficient HCV vaccine using Gel.NPs as a delivery system. Gel.NPs were prepared with optimum particle size and high positive zeta charge. *NS2* gene was amplified from the Egyptian HCV genotype 4a (isolate ED43), then successfully cloned and expressed in E.coli using pQE-30 vector. The antigenicity of the recombinant NS2 protein was confirmed when reacted with sera from patients with HCV. *NS2* and Gel.NPs were conjugated and the *NS2*/Gel.NPs conjugate showed higher efficiency in bacterial transformation than *NS2* gene alone.

## Materials and methods

### Materials

Gelatine type B (gel strength ~300 g Bloom, Sigma-Aldrich, US), Glutaraldehyde (25%, Sigma-Aldrich, US), Acetone (99.9%, Sigma-Aldrich, US), E.coli M15 (Qiagen Inc., Valencia, USA). LB broth Miller (Luria-Bertani, Amresco,US), Ampicillin (Sigma-Aldrich, US), Kanamycin (Sigma-Aldrich, US), Isopropyl b-D-thiogalactoside (IPTG) (≥99% (TLC), ≤0.1% Dioxane, Sigma-Aldrich, US), PBS (Sigma, US), PMSF (Sigma, Ltd. Dorset, England), 8 M urea (Sigma, US), 20 mM β-mercaptoethanol (β-ME) (Sigma-Aldrich,US) Ni2+/nitrilotriacetate (NTA)–agarose (Qiagen Inc., Valencia, USA), Bovine serum albumin (Sigma, US), Tris-buffered saline (Sigma, US), Horseradish peroxidase (HRP)-labeled Protein A (Sigma, Ltd. Dorset, England), ECL Plus^™^ Western Blotting Reagents from GE Healthcare (formerly Amersham Biosciences), Rhodamine red dye (PowerPlex^®^ 16 BIO System, Promega) and Quantifluor (QuantiFluor^®^ dsDNA System, Promega), High Pure PCR Purification Kit (Biobasic Inc., Ontario, Canada), QIAexpressionest kit (Qiagen Inc., Valencia, USA), Proteins and Molecular weight standards (Sigma-Aldrich, US), Nitrocellulose membranes (Wattman, US), QIAGEN EZ Competent Cells (Qiagen Inc., Valencia, USA), QIAprep SpinMiniprep kit (Qiagen Inc., Valencia, USA), SphI and HindIII (Roche), QIAGEN^®^ PCR Cloning kit (Qiagen Inc., Valencia, USA), GoTaq^®^ Green Master Mix (Promega).

### Methods

#### Preparation and characterization of gelatine nanoparticles (Gel.NPs)

Gel.NPs were synthesized by the two step desolvation method, described by Coester and colleagues, [26] with some modifications in the temperature, pH, concentration of glutaraldehyde and the type of desolvating agent “S1 Table”. Briefly, one gram of gelatin type B was dissolved in 20 ml distilled water with gentle heating. For the first desolvation step; 20 ml acetone was added; gelatin fractions were precipitated after 15 min and the supernatant was discarded. To dissolve the precipitate; 20 ml water was added under gentle heating at pH 3 that was adjusted using 0.1 N hydrochloric acid (HCL). In situ, Gel.NPs were formed during the second desolvation step by dropwise addition of 80 ml acetone while stirring (500 rpm). After 10 min, 100 μl of 25% glutaraldehyde were added to crosslink the nanoparticles under stirring for 12 h. Gel.NPs were purified by three-fold centrifugation (16000 g for 20 min) in 30% acetone. Purified Gel.NPs were dried, suspended in a highly purified water (conductivity < 0.04 μs/cm) and stored at 4–8°C.

Physico-chemical properties of Gel.NPs were characterized using high-resolution transmission electron microscope (HR-TEM, FEI, Tecnia G20, Eindhoven, and Netherland), X-ray Diffraction (XRD, PanAnalytical, X’pert Pro, Almelo, Netherland) and particle size analyzer (Zeta sizer a nano series s’, Malvern, Worcestershire, UK) “S1–S3 Figs”.

#### Amplification and cloning of *NS2* gene

*NS2* gene was amplified from the recombinant HCV genotype 4a (isolate ED43) genome (GenBank accession number: Y11604) (kindly provided by Dr. Richard Eliott lab, Institute of Virology, University of Glasgow, Church Street, Glasgow). The amplification of *NS2* gene (650 bp, extends from 2707 to 3357 bp of the genome) was performed as mentioned in the manufacturer’s manual of the PCR master mix. *SphI and HindIII* restrictions sites (found to have zero cuts using the online program NEB cutter version 2.0 (nc2.neb.com/NEBcutter2) have been added at the 5’end of the forward and reverse primers respectively, and their sequences were as follows: 5' GCATGCTACGACCAGGAAGTGGCAGG –3', and 5'- AAGCTTAAGGAGTCTCCACCCCTTTGA -3'. PCR program consisted of 30 cycles with initial denaturation 95°c for 5 min, denaturation 95°c for 30 sec, annealing 55°c for 45 sec, extension 72°c for one min, and final extension72°c for 10 min. The *NS2* amplicon was purified from gel using High Pure PCR Purification Kit (Biobasic Inc., Ontario, Canada) [27] as per manufacturer’s instructions, double digested with *SphI* and *HindIII* and repurified Then, *NS2* was cloned using QIAGEN^®^ PCR Cloning kit and the steps were performed as mentioned in the manufacturer’s manual, with the only modification in using pQE-30 cloning and expression vector instead of pDrive cloning vector provided with the kit in order to perform both cloning and expression with the same construct. Recombinant *NS2* concentration and purity were measured using UV spectrophotometer (Q5000, Quawell, USA)

#### Expression of recombinant *NS2* and antigenicity

The pQE-30 vector contains the sequence for the expression of N-terminally 6xHis-tagged proteins, and it also provides the multiple cloning sites in the first reading frame.

After cloning; Recombinant *NS2* was transformed into M15 competent cells using QIAexpressionest kit (Qiagen Inc., Valencia, USA). Bacteria were grown overnight at 37°C and the recombinant hexahistidine-tagged *NS2* protein was purified under denaturing conditions on Ni2+-nitrilotriacetate (NTA)–agarose (Qiagen Inc., Valencia, USA) according to the manufacturer’s instructions. *NS2* recombinant protein was detected by electrophoresing denaturing SDS-PAGE acrylamide gel which was then stained with coomassie blue.

The antigenicity of *NS2* protein was then investigated by Western blotting. Briefly, NS2 protein was electrophoresed in 12% SDS-PAGE acrylamide gels and transferred onto nitrocellulose membranes (Wattman, US). Membranes were then blocked with 1% bovine serum albumin in Tris-buffered saline for 2 h at room temperature with shaking. Then, the membranes were incubated with anti-HCV human sera of Egyptian blood donors infected with hepatitis C, washed, incubated with horseradish peroxidase (HRP)-conjugated second antibody for 1 h at 37°C and the membrane was developed using the enhanced chemiluminescent (ECL^®^) kit following the manufacturer’s instructions. The same steps were repeated using sera from healthy Egyptian donors to ensure the specificity of the recognized protein.

#### Conjugation of the recombinant *NS2* gene with Gel.NPs

Gel.NPs were labeled with Rhodamine red (0.001g of Gel.NPs dissolved in 400μl dH_2_O with 2μl Rhodamine red dye). The recombinant *NS2* gene was labeled with fluorescein dye (1μl *NS2* gene + 199μl Quantifluor), then excited with the corresponding laser lines, 633 nm for Rhodamine red dye (red color) and 514 nm for Quantifluor dye (green color).

Recombinant NS2 concentration was estimated to be 50 ng/μl by UV spectrophotometer and the final concentration of Gel.NPS after labelling was 2500 ng/μl. To prepare recombinant *NS2*/Gel.NPs conjugates; different ratios have been tried but the ratios 1:50, 2:50 and 1:100 (w/w) of *NS2* gene and the labeled Gel.NPs respectively have been used in further experiments as they have resulted in successful and consistent transformation. For this purpose; labeled *NS2* gene and Gel.NPs were co-incubated over one day on 500 rpm shaker to form *NS2*/Gel.NPs conjugate by physical conjugation. To estimate the amount of recombinant NS2 bound to Gel.NPs, NS2/Gel.NPs conjugate was precipitated by three-fold centrifugation at 16000 g for 20 min (till the purity of DNA in supernatant reached 1.8–2), the amount of recombinant *NS2* was measured in the supernatant by UV spectrophotometer (Q5000, Quawell, USA), then the amount of recombinant *NS2* conjugated to Gel.NPs was estimated as previously described (Singh and Mishra, 2014). To get rid of the excess unbound *NS2* gene, the supernatant was discarded and the conjugated *NS2*/Gel.NPs were resuspended in deionized water.

Confocal Laser Scanning Microscope (CLMS) imaging was utilized to validate the conjugation process between recombinant *NS2* gene and Gel.NPs.

#### Transformation of *NS2*/Gel.NPs into DH5-α bacteria

Transformations of the labeled Gel.NPs, *NS2* gene, and the *NS2* /Gel.NPs conjugate into DH5-α bacteria were assessed using four replicates for each sample. CLSM technique was used to study the efficiency of Gel.NPs to deliver *NS2* gene into DH5-α bacteria compared to *NS2* gene alone; one colony from the transformed DH5-α bacteria was picked from each sample, fixed on the slide by 20% glutaraldehyde and excited using the suitable laser as described in Table 1. For quantitative analysis, bacterial replicates were counted using ImageJ software (National Institutes of Health, Bethesda, MA, USA).

**Table 1:** Different laser wavelengths used to detect prepared samples.

Sample	Type of excited laser	
labeled Gel.NPs	633nm	
labeled recombinant *NS2* gene	405nm for gene that absorb at UV region	514nm for Quantifluor dye labeling gene
Conjugate of labeled (Gel.NPs+ recombinant *NS2* gene)	633nm,405nm and 514nm	

Finally, in order to confirm that Gel.NPs didn’t affect the recombinant *NS2* gene structure, minipreps of the transformed bacteria with the three tested concentrations were prepared using QIAprep SpinMiniprep kit (Qiagen Inc., Valencia, USA) following the manufacturer instructions, in which a single colony from each plate was picked, inoculated into 5 ml of LB containing 5μl ampicillin (LBAmp broth) and grown overnight at 37°C.

## Results

### Characterization of gelatin nanoparticles

**Fig 1:**
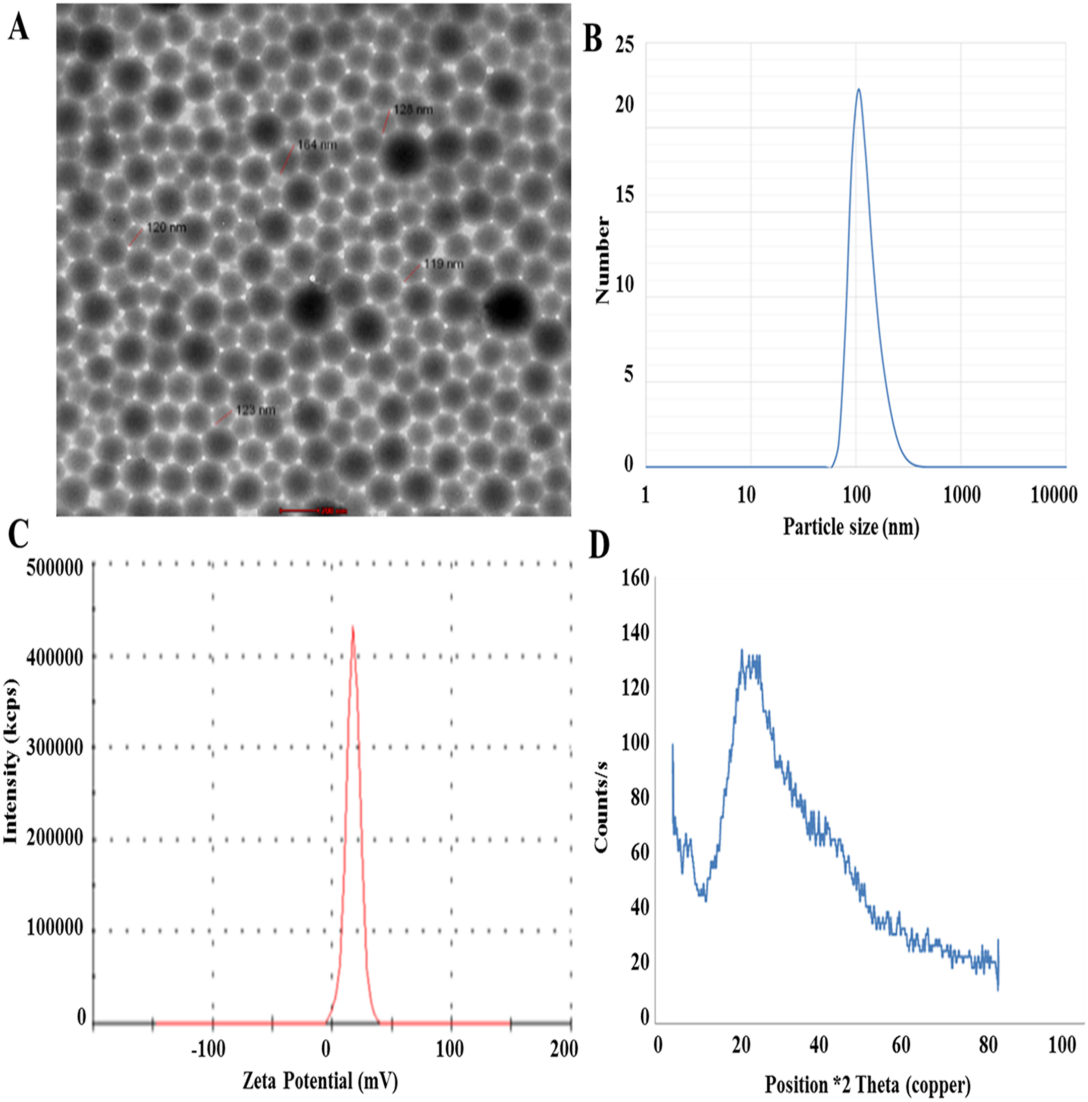
Characterization of Gel.NPs. **A**. HR-TEM image of the prepared Gel.NPs resuspended in water and adsorbed onto solid support and stained with phosphotangestic acid which shows that particles have well dispersed spherical shape with average particles. **B**. Graph showing particle size distribution by number for the synthesized Gel.NPs that was obtained by particle size analyzer. As shown from the observed peak the size of Gel.NPs is 150±2 nm. C. Graph showing the Zeta potential for synthesized Gel.NPs that was determined by measuring the electrophoretic mobility of the Gel.NPs using a Malvern zeta sizer. As shown the observed Zeta potential is +17.6 mV. D. XRD of prepared GNPs.

Morphological characterization of Gel.NPs showed nearly perfect unitized and well dispersed spherical particles with an average size of 150.0 ± 2.0 nm, using high-resolution TEM. These observations were supported by particle size analysis done by Dynamic Light Scattering (DLS) technique (Fig 1A). The particle size was measured showing to have an average size of 150 nm with low Polydispersity Index (PDI) 0.09 which indicates its narrow size distribution (Fig 1B). Zeta-Potential (surface charge) of the prepared Gel.NPs was improved to be more electro-positive (+17.6 mV) (Fig 1C). Phase analysis was performed using X-Ray Diffraction technique (XRD) (Fig 1D). XRD pattern showed a characteristic broad peak at angle θ = 20° indicating that the prepared Gel.NPs have an amorphous structure.

### Cloning and Expression of the *NS2* gene

**Fig 2:**
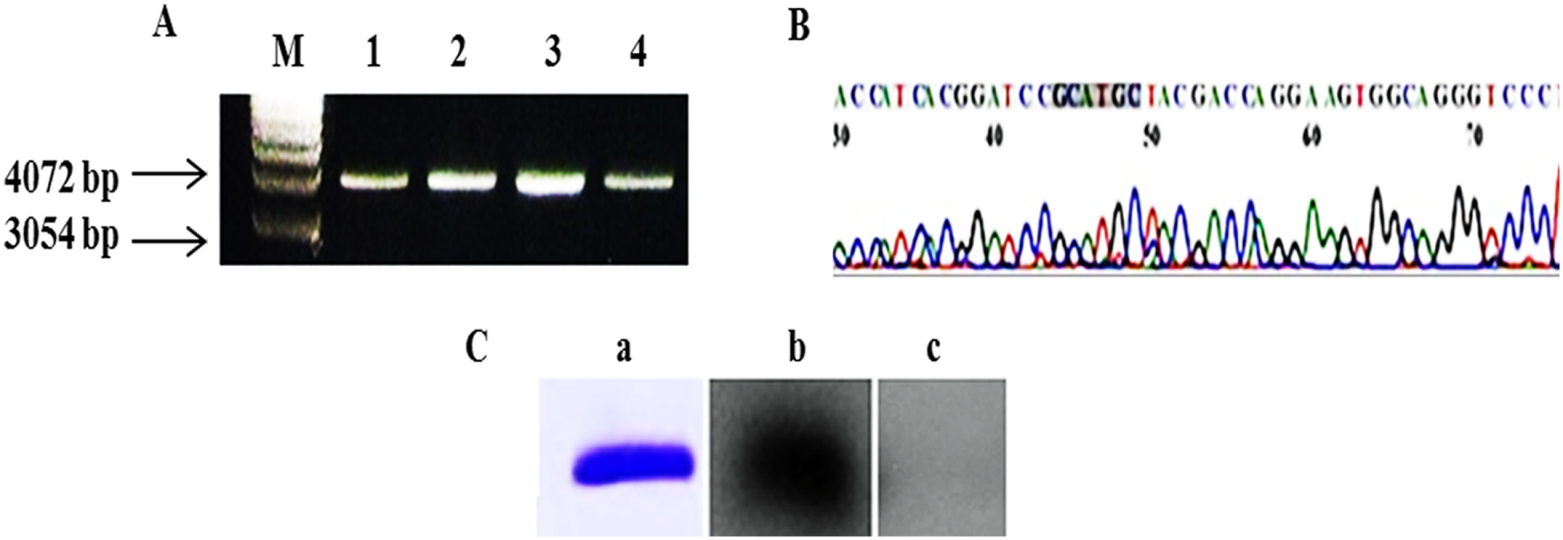
Cloning and expression of *NS2* gene in pQE30. **A**. Agarose gel electrophoresis for single digestion of the miniprep of the recombinant NS2 samples with *SphI*. Lane M: 1 kb DNA marker, Lane 1–4: DNA resulted from minipreps of four different clones digested with *SphI* showing a band of ~ 4 kb indicating *NS2* insert (650 bp) within the pQE-30 vector (3.4 kb). **B**. Representative DNA sequence analysis of the *NS2* insert into pQE-30 vector using ABI PRISM model 310 DNA automated sequencer. Bases 1–50 are pQE-30 vector sequence, bases from 50 to the end are partial *NS2* sequene. Shadowed sequence is the restriction site for *SphI*. **C**. **a**: SDS-PAGE of M15 bacterial lysate resulted from expression of the *NS2* protein Lane M: prestained wide range MW marker. Lane 1: expressed *NS2* protein after purification. **b**: Western blot of the purified *NS2* protein recognized by sera of Egyptian patients infected with HCV. **c**: Western blot for healthy donors showed no specific recognition of the *NS2* protein.

*NS2* gene was successfully amplified, cloned and expressed into protein and the antigenicity of *NS2* protein was confirmed by Western blotting in which a band of ~23 kDa corresponding to the expressed *NS2* protein was recognized by anti-HCV antibodies present in the sera of Egyptian patients infected with HCV, while healthy donors showed no specific recognition of the *NS2* protein (Fig 2).

### Conjugation of recombinant *NS2* with Gel.NPs

**Fig 3:**
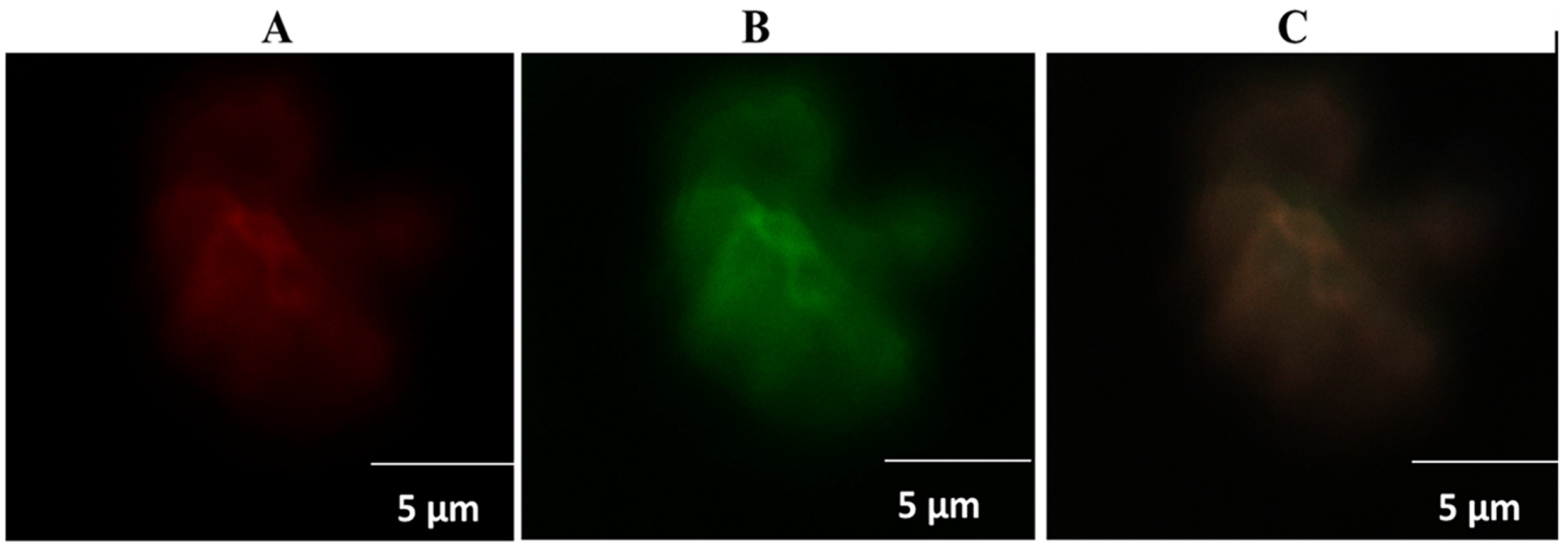
Confocal Laser Scanning Microscope (CLSM) imaging. **A**: Gel.NPs labeled with for Rhodamine red dye excited at 633 nm. **B**: *NS2* gene labeled with Quantifluor exited at 514 nm. **C**: labeled *NS2* /Gel.NPs conjugate excited simultaneously at both 633 nm and 514 nm.

It was very important to estimate the amount of the recombinant *NS2* gene that gets conjugated to Gel.NPs and to confirm the conjugation between *NS2* gene and Gel.NPs. We found that *NS2*/Gel.NPs with ratio of 1:100 have shown the highest conjugation percentage as 62.6% of the recombinant *NS2* gene were conjugated to Gel.NPs, while 43%, 44.2% of the recombinant *NS2* gene in *NS2*/Gel.NPs with ratios of 1:50, 2:50 respectively were successfully conjugated “S2 Table”. Then, confirmation of conjugation was carried out using CLMS technique. Excitation of Gel.NPs labeled with Rhodamine red at 633 nm showed highly intense red aggregations of gelatin nanoparticles, (Fig 3A). *NS2* gene labeled with Quantifluor was exited at 514 nm laser line showed green aggregations of *NS2* gene, (Fig 3B). While simultaneous excitation of *NS2*/Gel.NPs conjugate with both laser lines (633 nm and 514 nm) showed yellowish-orange clusters as a result of the crossing over between the two laser dyes Rhodamine (red) and Quantifluor (green), corresponding to the conjugation between the Gel.NPs and the *NS2* gene and confirm the generation of the new *NS2*/Gel.NPs conjugate that can be used as a simple and direct gene delivery system, (Fig 3C).

### Gelatin nanoparticles enhanced NS2 transformation into E. coli

**Fig 4:**
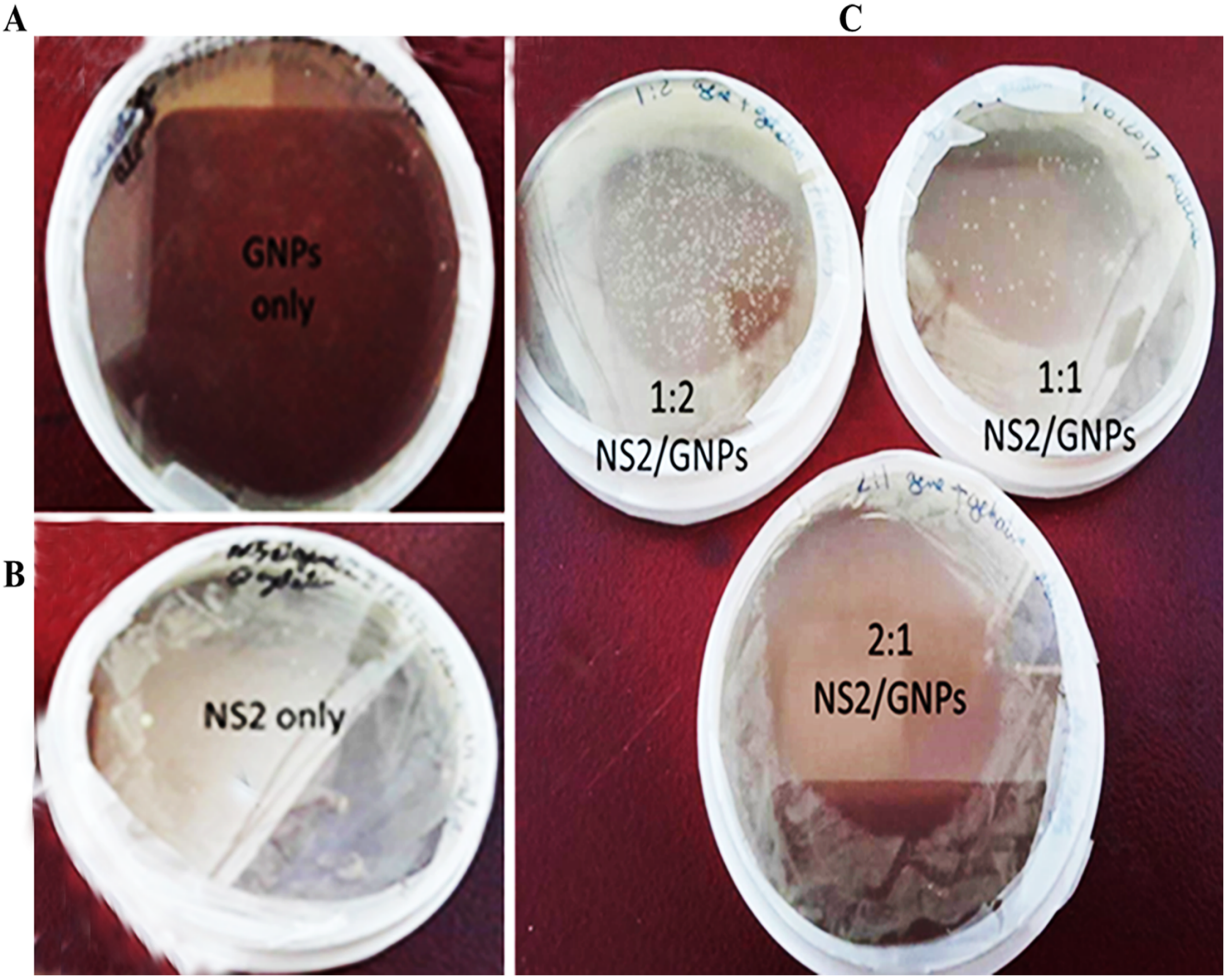
Images of plates containing DH5-α bacteria transformed with A) Gel.NPs only, B) recombinant *NS2* gene only, C) labeled conjugate recombinant *NS2* Gel.NPs with three ratios (w/w) 1:50, 2:50 and 1:100.

**Fig 5:**
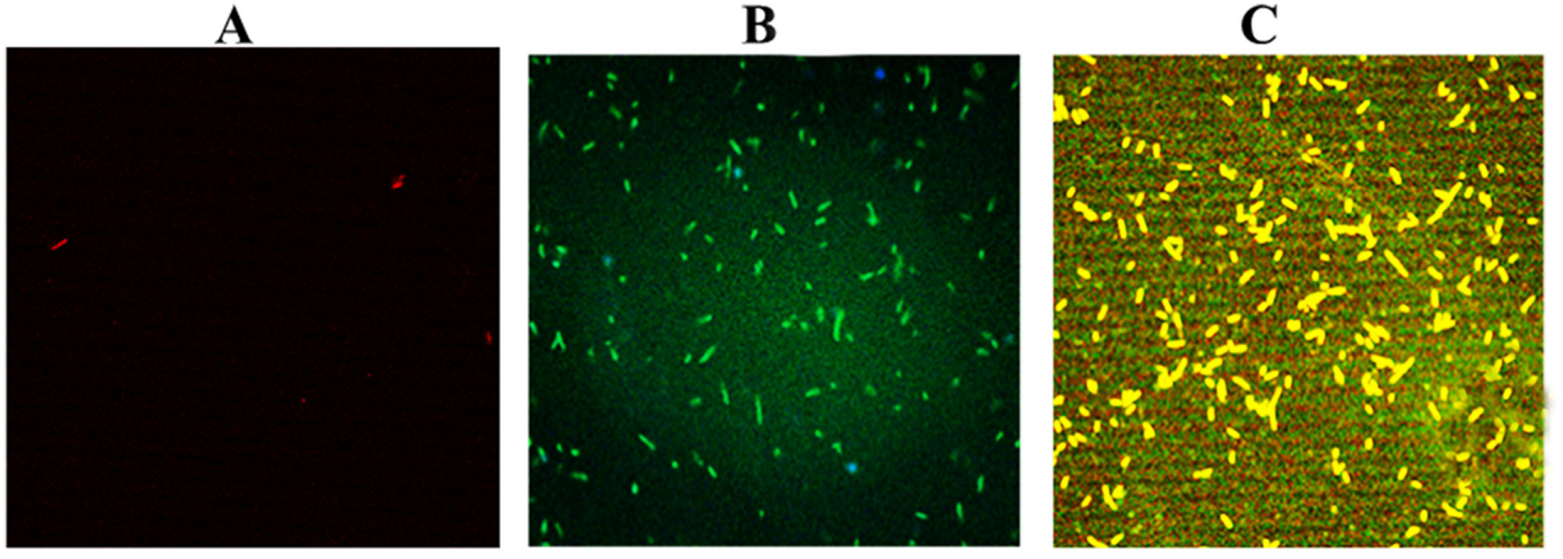
The image of *NS2*/Gel.NPs transformed DH5-α bacteria by Confocal Laser Scanning Microscope showed high number of colonies in yellow-orange color after laser excitation at 633 nm and 514 nm simultaneously. The image of Gel.NPs transformed into DH5-α bacteria showed no appearance of any emission colors due to absence of plasmid carrying ampicillin resistant gene, where bacteria died when grown in presence of ampicillin (Fig 5A). *NS2* transformed DH5-α bacteria showed lower number of bacterial colonies in green color after laser excitation at 514nm (Fig 5B). The yellow-orange color due to the cross over between the red labeled Gel.NPs and green labeled *NS2* gene could be detected in (Fig 5C) which indicates that presence of gelatin nanoparticles enhance transformation efficiency than using naked *NS2* in (Fig 5B).

In order to evaluate the efficiency of gelatin nanoparticles as a delivery system for the recombinant *NS2* gene, three labeled recombinant *NS2*/Gel.NPs conjugate samples ratios (w/w); 1:50, 2:50 and 1:100, in addition to the labeled *NS2* gene alone and the labeled Gel.NPs samples alone were transformed into DH5-α bacteria which were cultured for 24 h under optimum conditions. Interestingly, the highest transformation efficiency was observed in the plates of the recombinant labeled *NS2*/Gel.NPs conjugate, (Fig 4C), compared to the plates of labeled *NS2* gene alone, (Fig 4B), while no transformation could be detected with the labeled Gel.NPs only (Fig 4A). Moreover; the highest number of bacterial colonies was observed in the plate with bacteria transformed with recombinant *NS2*/Gel.NPs conjugate with ratio 1:100 compared with the other ratios. The high efficiency of transformation of *NS2* conjugated to Gel.NPs was confirmed by imaging bacterial cells using Confocal Laser Scanning Microscope and the number of bacterial replicates was counted using ImageJ software. The results showed that the number of transformed bacterial replicates with *NS2*/Gel.NPs (1:100) was higher (236± 3.82) (Fig 5C) than other used ratios and than those with *NS2* alone (119±3.03) (Fig 5B) and those with Gel.NPs alone (no bacterial growth) (Fig 5A) “S3 and S4 Tables”, “S4 Fig”.

**Fig 6:**
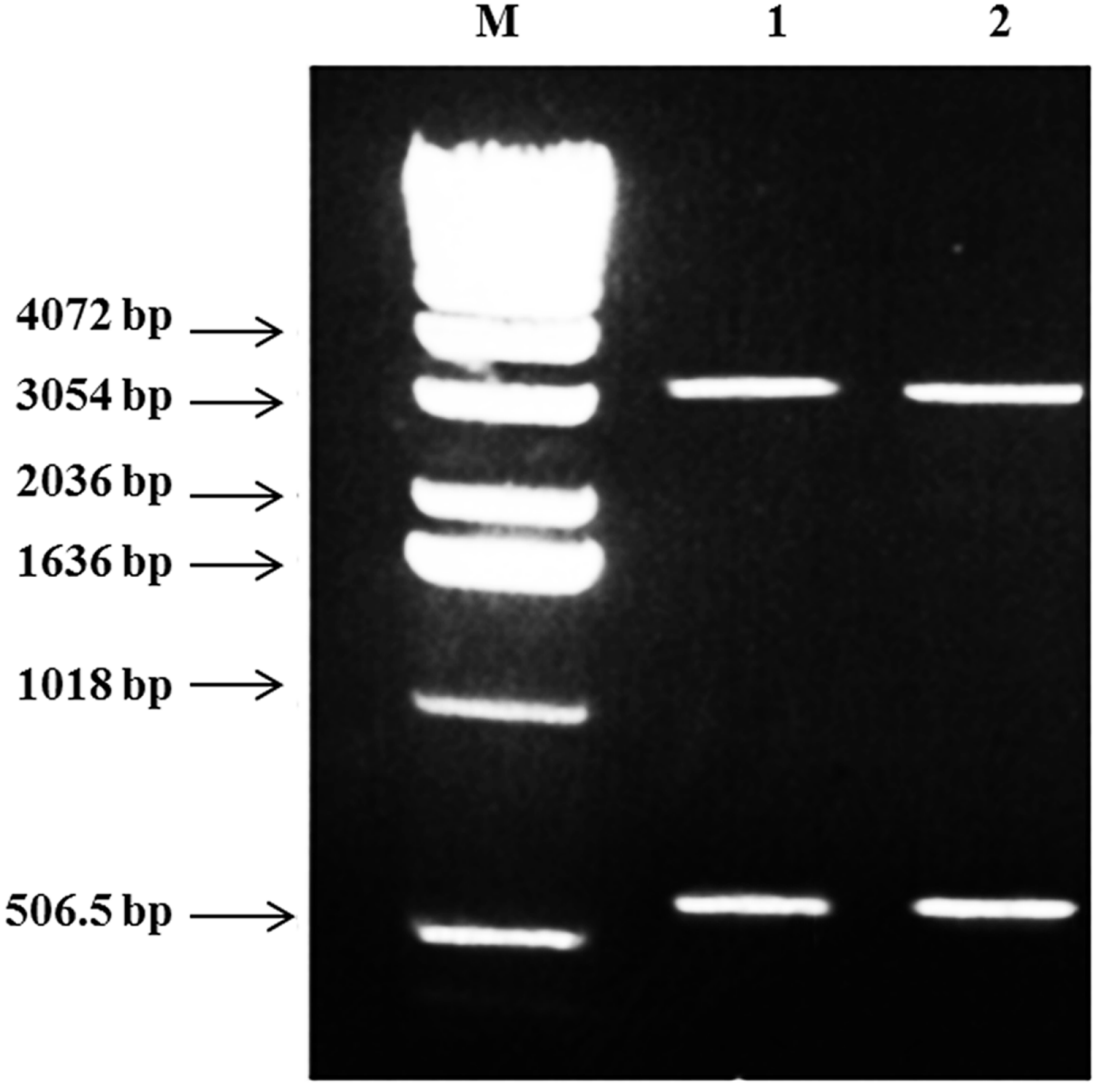
Agarose gel electrophoresis for miniprep samples of the recombinant PQE 30/*NS2* plasmid/Gel.NPs double digested (*SphI*/*HindIII*) showing two bands; band for *NS2* insert (650 bp) and band for pQE-30 vector (3.4 kb) in Lane 1 and 2. Lane M: 1 kb DNA marker.

Finally, gel electrophoresis of the miniprep product of the transformed bacteria confirmed that the Gel.NPs didn’t affect the recombinant *NS2* gene structure (Fig 6), where the two bands appeared for *NS2* insert (650 bp) and the pQE-30 vector (3.4 kb) were exactly at the correct size.

## Discussion

*In vitro* and *in vivo* applications of gelatin in the controlled release devices for bioactive molecules like proteins or plasmid DNA were reviewed [24], and the scientific contributions of gelatin nanoparticles as an ideal carrier system for drug delivery have been increased in the last five years [28–30]. In the present study, we investigated the effectiveness of Gel.NPs as an antigen delivery system against HCV. A previous study has demonstrated that Gel.NPs incorporated in the bronchial epithelial cells and 16HBE14o-cells showed only a few or no cytotoxicity without any inflammatory responses [31]. These findings suggest that Gel.NPs may be a suitable and safe candidate as drug or gene carriers.

Many studies have reported several methods for Gel.NPs synthesis [26,32,33]. Here in, we applied some different processing parameters such as pH, the amount of glutaraldehyde added, and temperature, these variations resulted in synthesis of nanoparticles with particle size and shape smaller than the earlier published work [26,32,33]. Our preparation conditions were optimized using 100μl /gm of glutaraldehyde (GTA) at pH 3, that resulted in synthesis of well dispersed spherical particulate (150±10 nm), which is smaller in size than the previously synthesized gelatin nanoparticles using COESTER method that is used as a reference for all consequent preparations in many studies and usually results in nanoparticles with bigger size (~280 nm) [26]. Previously, cationic Gel.NPs have shown to increase the stability of the nanoparticle system and improve the interaction between the particles and the eye surface of the rabbits and thus increased the potential of ocular drug delivery more than negatively charged Gel.NPs [34]. In addition, cationized Gel.NPs have been proved to act as a highly effective carrier system for immunogenic CpG oligodeoxynucleotides due to its protein based structure that offers many functional groups that can help as anchor of an antigen [35].

*In vitro* trials have been carried out for using *NS2* gene as HCV vaccine but the transformation efficiency still the main challenge. In the present work, we introduced the gelatin nanoparticles as a vehicle for *NS2* to facilitate and increase the transformation efficiency as a preliminary step in order to be used as an HCV vaccine delivery system. Stability of our prepared cationic Gel.NPs was improved at +17.6 Mv. compared to -23.1 Mv as mentioned in a previous study [36]. This stability might have played an important role in maintaining the stable electrostatic interaction between Gel.NPs and the negatively charged recombinant *NS2* gene to form the intended *NS2*/Gel.NPs conjugate, in addition to facilitate the penetration of the conjugate into the negatively charged cell membrane and nucleus of the target bacteria which is assumed to enhance the gene delivery process more than the gene alone [34].

Successful transformation was confirmed by imaging the labeled Gel.NPs, the labeled recombinant *NS2* gene and the labeled conjugate recombinant *NS2/*Gel.NPs samples using Confocal Laser Scanning Microscope (CLMS), thus proving that Gel.NPs can be used as a successful gene delivery system into the bacteria as a primary step for testing it later on the human cell line level. To test the transformation efficiency of Gel.NPs, different concentrations of Gel.NPs were used as vehicles to carry the recombinant *NS2* gene for intracellular delivery of the DH5-α bacteria, the numbers of transformed bacterial colonies were counted by ImageJ software (National Institutes of Health, Bethesda, MA,USA) after imaging. The present data revealed that recombinant *NS2/*Gel.NPs conjugated with ratio 1:100induced the highest transformation yield compared to the transformation of *NS2* gene alone, thus indicating that Gel.NPs increased the transformation efficiency. Gel.NPs might have protected the recombinant DNA from intracellular degradation or digestion as has been proved in a previous study in which Vu L. Truong-Le and his colleagues demonstrated that DNA conjugated to Gel.NPs is more resistant to nuclease digestion than naked DNA [37]. We could attribute the enhancement in transformation efficiency at 1:100 rather than the other used ratios to the increase in cationic Gel.NPs concentration which in turn increased the possibility of conjugating higher number of negatively charged *NS2* molecules [38]. Furthermore, cationic Gel.NPs have more potential to interact with the cell surface and nuclear membrane and thus increased the transfection efficiency [34].

In order to investigate the effect of Gel.NPs on the recombinant DNA, we repeated the Miniprep steps on the transformed bacteria and confirmed that Gel.NPs did not affect the recombinant *NS2* gene structure or size and consequently, would not impede *NS2* protein expression. Thus, we could implicate Gel.NPs as a safe and an efficient delivery system that can transport recombinant *NS2* gene without affecting its structure, and this indicates that *NS2*/Gel.NPs complex can be expressed successfully within the DH5-α bacterial cell.

## Conclusion

Gel.NPs were successfully synthesized with optimum controlled characteristics, conjugated with recombinant *NS2* gene and facilitated the delivery of the *NS2* gene into the bacterial cell *in vitro* effectively where it could be amplified and expressed in the bacterial cell without affecting its structure. This study suggests that Gel.NPs could be a useful vaccine and gene delivery system and may be used in conjugation with the *NS2* gene for HCV treatment. However, further investigations are required to shed light upon the validity of using such a conjugate as a vaccine against HCV *in vivo*.

## Supporting information

S1 Fig TEM images of Gelatin nanoparticles prepared by three methods, A: with 400μl glutaraldehyde and pH 7; B: with 200μl glutaraldehyde and pH3.6; C: with 100μl glutaraldehyde and pH3. The TEM image of the first preparation procedure (S1 Table; **Method 1**) showed that these particles have semi spherical shape with average size of 423 nm; illustrated in **(S1 Fig A)**. The TEM image of the second preparation procedure (S1 Table; **Method 2**) showed that these particles have aggregate complex shape with average size of 350 nm; illustrated in **(S1 Fig B)**. The TEM image of the third preparation procedure (S1 Table; **Method 3**) showed that these particles have spherical shape with average size of 150.0 ± 2.0 nm; illustrated in **(S1 Fig C)**.
(DOCX)

S2 Fig Particle size of Gel.NPs prepared by three methods, A: Gel.NPs with particle size 423 nm; B: Gel.NPs with particle size 350 nm; C: Gel.NPs with particle size 150 nm. Particle size distribution measurements was in accordance with TEM imagine, where Particle size image using (Malvern Instruments, UK) of the prepared Gel.NPs (S1 Table; **Method 1**) showed that these particles have average size of 423 nm with polydispersity index 1.00; illustrated in **(S2 Fig A)**. Particle size image of the prepared Gel.NPs (S1 Table; **Method 2**) showed that these particles have average size of 350 nm with polydispersity index 0.294; illustrated in **(S2 Fig B)**. Particle size image of the prepared Gel.NPs (S1 Table; **Method 3**) showed that these particles have average size of 150 nm with polydispersity index 0.109; illustrated in **(S2 Fig C)**.
(DOCX)

S3 Fig Zeta potential of Gel.NPs prepared by three methods, A: zeta potential +0.3mV; B: zeta potential -21mV; C: zeta potential +17.6 mV. It was very important to get information about the capping charges around the Gel.NPs particles where the more positivity the more stability, Zeta potential measurement were done to evaluate the particle surface charge, where Zeta potential using (Malvern Instruments, UK) of the prepared Gel.NPs (S1 Table; **Method 1**) showed that these particles have +0.3 mV; illustrated in **(S3 Fig A)**. Zeta potential of the prepared Gel.NPs (S1 Table; **Method 2**) showed that these particles have -21 mV; illustrated in **(S3 Fig B)**. Zeta potential of the prepared Gel.NPs (S1 Table; **Method 3**) showed that these particles have +17.6 mV; illustrated in **(S3 Fig C)**.
(DOCX)

S4 Fig Number of bacterial replicates transformed with *NS2* gene+Gel.NPs, *NS2* gene alone and Gel.NPs alone. ImageJ software was used to count bacterial replicates in confocal micrographs. Each bar represents mean ± SE of four independent experiments. As shown the number of bacterial colonies in the plate with bacteria transformed with recombinant *NS2*+Gel.NPs conjugate was higher than the number of bacterial colonies in the plate with bacteria transformed with recombinant *NS2* gene alone. While no bacterial colonies in the plate with bacteria transformed with Gel.NPs alone.
(DOCX)

S1 Table Evaluation of optimized conditions for the reproducible preparation of distinctive Gelatin nanoparticles (Gel.NPs). (DOCX)

S2 Table UV spectrophotometer measurements to estimate the amount of recombinant NS2 bound to Gel.NPs. (DOCX)

S3 Table Raw Data for number of bacterial replicates transformed with *NS2* gene+Gel.NPs, *NS2* gene alone and Gel.NPs alone. ImageJ software was used to count bacterial replicates in Confocal Laser Scanning Micrograph in Fig 5. Four plates were prepared for each type of transformation and confocal micrograph was captured for each plate.
(DOCX)

S4 Table Number of bacterial replicates transformed with *NS2* gene+Gel.NPs, *NS2* gene alone and Gel.NPs alone. ImageJ software was used to count bacterial replicates in confocal micrographs.
(DOCX)
