# Supplementary material for: Gelatin nanoparticles enhance delivery of hepatitis C virus recombinant NS2 gene
Source: PLoS One. 2017 Jul 26;12(7):e0181723. doi: 10.1371/journal.pone.0181723 (PMC5528829; doi:10.1371/journal.pone.0181723)
Supplement: S1 Fig — The TEM image of the first preparation procedure (S1 Table; Method 1) showed that these particles have semi spherical shape with average size of 423 nm; illustrated in (S1 Fig A). The TEM image of the second preparation procedure (S1 Table; Method 2) showed that these particles have aggregate complex shape with average size of 350 nm; illustrated in (S1 Fig B). The TEM image of the third preparation procedure (S1 Table; Method 3) showed that these particles have spherical shape with average size of 150.0 ± 2.0 nm; illustrated in (S1 Fig C). (DOCX) [file pone.0181723.s001.docx]

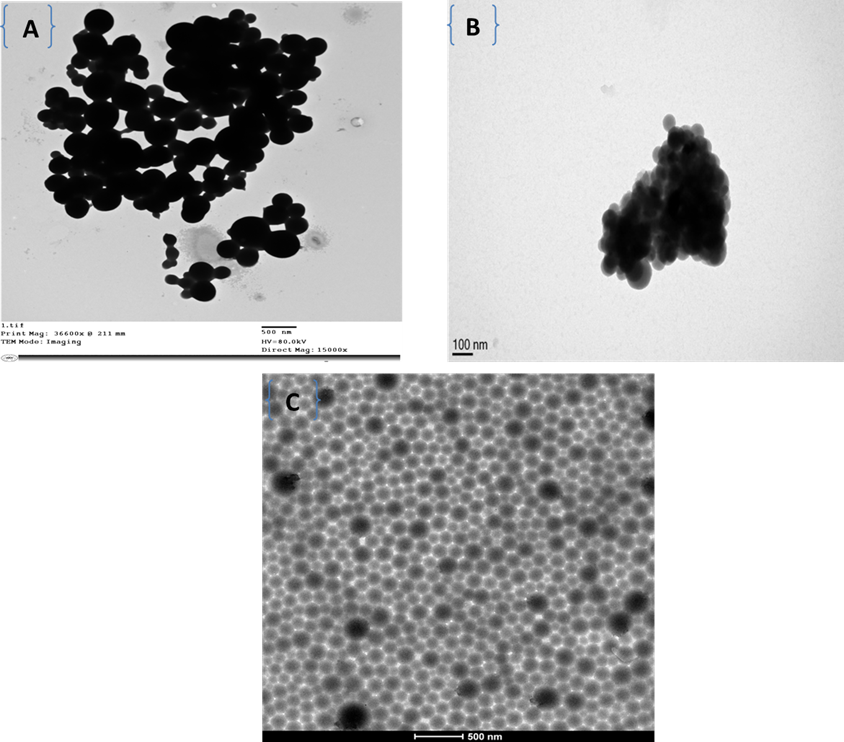


**S1 Fig.** TEM images of Gelatin nanoparticles prepared by three methods, **A:** with 400µl glutaraldehyde and pH 7; **B:** with 200µl glutaraldehyde and pH3.6; **C:** with 100µl glutaraldehyde and pH3. The TEM image of the first preparation procedure **(S1 Table; Method 1)** showed that these particles have semi spherical shape with average size of 423 nm; illustrated in **(S1 Fig** **A)**. The TEM image of the second preparation procedure **(S1 Table; Method 2)** showed that these particles have aggregate complex shape with average size of 350 nm; illustrated in **(S1 Fig** **B).** The TEM image of the third preparation procedure **(S1 Table; Method 3)** showed that these particles have spherical shape with average size of 150.0 ± 2.0 nm; illustrated in **(S1 Fig C).**
