# Supplementary material for: Gelatin nanoparticles enhance delivery of hepatitis C virus recombinant NS2 gene
Source: PLoS One. 2017 Jul 26;12(7):e0181723. doi: 10.1371/journal.pone.0181723 (PMC5528829; doi:10.1371/journal.pone.0181723)
Supplement: S2 Fig — Particle size distribution measurements was in accordance with TEM imagine, where Particle size image using (Malvern Instruments, UK) of the prepared Gel.NPs (S1 Table; Method 1) showed that these particles have average size of 423 nm with polydispersity index 1.00; illustrated in (S2 Fig A). Particle size image of the prepared Gel.NPs (S1 Table; Method 2) showed that these particles have average size of 350 nm with polydispersity index 0.294; illustrated in (S2 Fig B). Particle size image of the prepared Gel.NPs (S1 Table; Method 3) showed that these particles have average size of 150 nm with polydispersity index 0.109; illustrated in (S2 Fig C). (DOCX) [file pone.0181723.s002.docx]

**
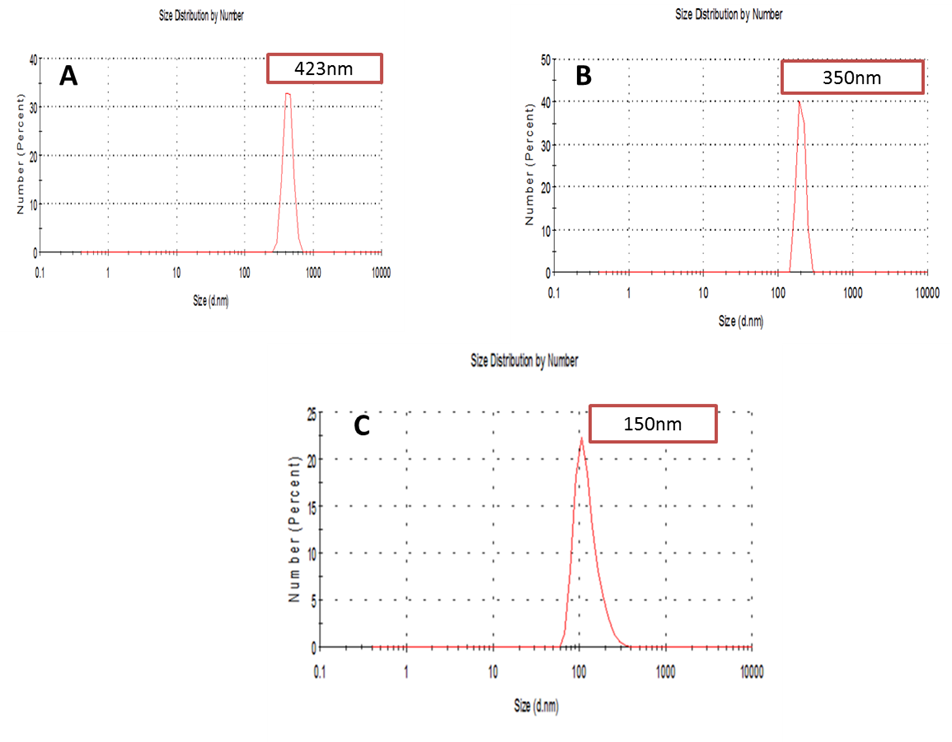
**

**S2 Fig.** **Particle Size of Gel.NPs prepared by three methods**, **A:** Gel.NPs with particle size 423 nm; **B:** Gel.NPs with particle size 350 nm; **C:** Gel.NPs with particle size 150 nm. Particle size distribution measurements was in accordance with TEM imagine, where Particle size image using (Malvern Instruments, UK) of the prepared Gel.NPs **(S1 Table; Method 1)** showed that these particles have average size of 423 nm with polydispersity index 1.00; illustrated in **(S2 Fig A).** Particle size image of the prepared Gel.NPs **(S1 Table; Method 2)** showed that these particles have average size of 350 nm with polydispersity index 0.294; illustrated in **(S2 Fig B).** Particle size image of the prepared Gel.NPs **(S1 Table; Method 3)** showed that these particles have average size of 150 nm with polydispersity index 0.109; illustrated in **(S2 Fig C).**
