# Supplementary material for: Gelatin nanoparticles enhance delivery of hepatitis C virus recombinant NS2 gene
Source: PLoS One. 2017 Jul 26;12(7):e0181723. doi: 10.1371/journal.pone.0181723 (PMC5528829; doi:10.1371/journal.pone.0181723)
Supplement: S3 Fig — It was very important to get information about the capping charges around the Gel.NPs particles where the more positivity the more stability, Zeta potential measurement were done to evaluate the particle surface charge, where Zeta potential using (Malvern Instruments, UK) of the prepared Gel.NPs (S1 Table; Method 1) showed that these particles have +0.3 mV; illustrated in (S3 Fig A). Zeta potential of the prepared Gel.NPs (S1 Table; Method 2) showed that these particles have -21 mV; illustrated in (S3 Fig B). Zeta potential of the prepared Gel.NPs (S1 Table; Method 3) showed that these particles have +17.6 mV; illustrated in (S3 Fig C). (DOCX) [file pone.0181723.s003.docx]

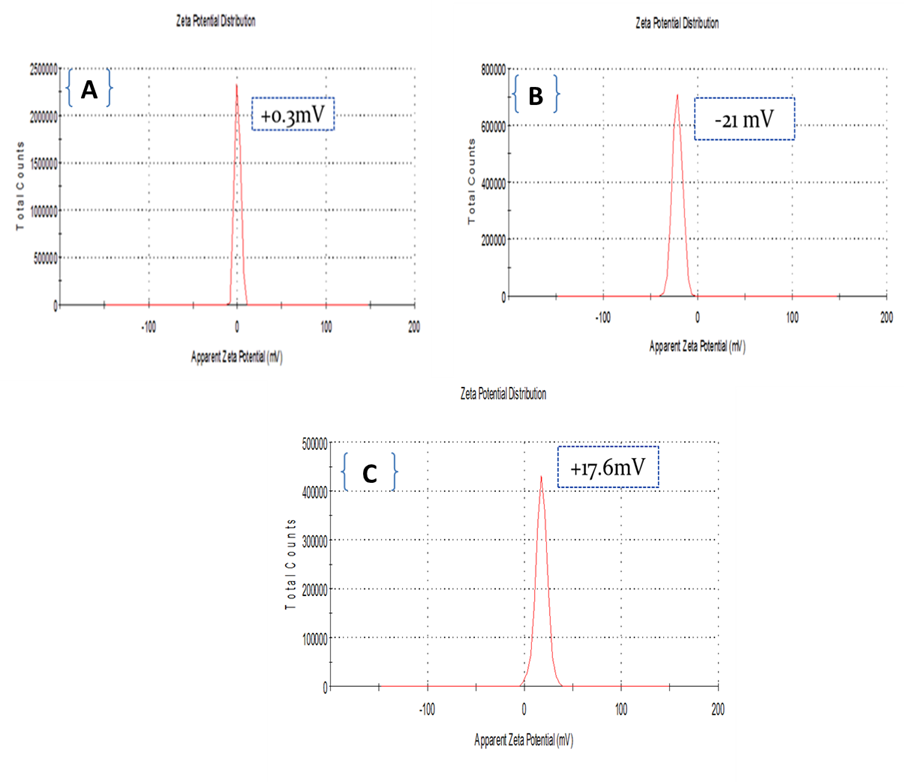


**S3 Fig. Zeta potential of Gel.NPs prepared by three methods**, **A:** zeta potential +0.3mV; **B:** zeta potential -21mV; **C:** zeta potential +17.6 mV. It was very important to get information about the capping charges around the Gel.NPs particles where the more positivity the more stability, Zeta potential measurement were done to evaluate the particle surface charge, where Zeta potential using (Malvern Instruments, UK) of the prepared Gel.NPs **(S1 Table; Method 1)** showed that these particles have +0.3 mV; illustrated in **(S3 Fig A)**. Zeta potential of the prepared Gel.NPs **(S1 Table; Method 2)** showed that these particles have -21 mV; illustrated in **(S3 Fig B)**. Zeta potential of the prepared Gel.NPs **(S1 Table; Method 3)** showed that these particles have +17.6 mV; illustrated in **(S3 Fig C).**
