# Supplementary material for: Gelatin nanoparticles enhance delivery of hepatitis C virus recombinant NS2 gene
Source: PLoS One. 2017 Jul 26;12(7):e0181723. doi: 10.1371/journal.pone.0181723 (PMC5528829; doi:10.1371/journal.pone.0181723)
Supplement: S4 Fig — ImageJ software was used to count bacterial replicates in confocal micrographs. Each bar represents mean ± SE of four independent experiments. As shown the number of bacterial colonies in the plate with bacteria transformed with recombinant NS2+Gel.NPs conjugate was higher than the number of bacterial colonies in the plate with bacteria transformed with recombinant NS2 gene alone. While no bacterial colonies in the plate with bacteria transformed with Gel.NPs alone. (DOCX) [file pone.0181723.s004.docx]

b

c

a

*

*

*

**S4 Fig. Number of bacterial replicates transformed with *NS2* gene+Gel.NPs, *NS2* gene alone and Gel.NPs alone**. ImageJ software was used to count bacterial replicates in confocal micrographs. Each bar represents mean ± SE of four independent experiments. As shown the number of bacterial colonies in the plate with bacteria transformed with recombinant *NS2*+Gel.NPs conjugate was higher than the number of bacterial colonies in the plate with bacteria transformed with recombinant *NS2* gene alone. While no bacterial colonies in the plate with bacteria transformed with Gel.NPs alone.

-There is a significant difference between groups by using one way ANOVA at P< 0.05 followed by Duncan multiple comparison test.

- The different letters means that there is a significant difference between groups by using Duncan multiple comparison test (P= 0.000).

-^*^ Statistically significant difference compared with other groups using student t- test (P= 0.000).
