# Supplementary material for: Gelatin nanoparticles enhance delivery of hepatitis C virus recombinant NS2 gene
Source: PLoS One. 2017 Jul 26;12(7):e0181723. doi: 10.1371/journal.pone.0181723 (PMC5528829; doi:10.1371/journal.pone.0181723)
Supplement: S1 Table — (DOCX) [file pone.0181723.s005.docx]

**S1 Table.** Evaluation of optimized conditions for the reproducible preparation of distinctive Gelatin nanoparticles (Gel.NPs).

| **Conditions:** | **Method 1** | **Method 2** | **Method 3** |
| --- | --- | --- | --- |
| Temp. before 1st desolvation (°C) | 40°C | 40°C | 50°C |
| Precipitation time(min.) | 30 min | 20 min | 15 min |
| Temp. before 2nd desolvation (°C) | 40 °C | 40 °C | 50 °C |
| pH before 2nd desolvation | 7 | 3.6 | 3 |
| speed of acetone addition  (2nd desolvation) (mL/min) | 1 ml/min | 1.5 ml/min | 3-5 ml/min |
| Amount of acetone  (2nd desolvation) | 40 ml | 40 ml | 40 ml |
| Amount of glutaraldehyde for crosslinking (25%) | 400 µl | 200 µl | 100 µl |
| **Particle size:** | **423 nm** | **350 nm** | **150 nm** |
| **Zeta potential:** | **+0.3 mV** | **-21 mV** | **+17.6 mV** |
| **Polydispersity Index:** | **1.00** | **0.294** | **0.109** |

According to **S1 Table**, the following standard parameters were chosen for nanoparticles preparation:

a) Temperature before the first and second desolvation step: 50 °C

b) Stirring speed: 500-700 rpm

c) Precipitation time after the first desolvation step: 15 min

d) Speed of acetone addition (second desolvation step): 3-5 ml/min

e) Amount of glutaraldehyde used for crosslinking: 100 µl
