# Supplementary material for: Gelatin nanoparticles enhance delivery of hepatitis C virus recombinant NS2 gene
Source: PLoS One. 2017 Jul 26;12(7):e0181723. doi: 10.1371/journal.pone.0181723 (PMC5528829; doi:10.1371/journal.pone.0181723)
Supplement: S2 Table — (DOCX) [file pone.0181723.s006.docx]

**S2 Table.** UV spectrophotometer measurements to estimate the amount of recombinant NS2 bound to Gel.NPs.

| Starting conc. of NS2 (ng) | conc. of NS2 in supernatant after conjugation to Gel.NPs (ng/µl) | Total conc. of NS2 in supernatant after conjugation to Gel.NPs | Conc. of NS2 conjugated to Gel.NPs ng (%) |
| --- | --- | --- | --- |
| 1500 | 19 | 855 ng/45 µl | 645 (43 %) |
| 1500 | 14 | 560 ng/40 µl | 940 (62.6) |
| 3000 | 41.85 | 1674 ng/40 µl | 1326 (44.2) |
