# Supplementary material for: Gelatin nanoparticles enhance delivery of hepatitis C virus recombinant NS2 gene
Source: PLoS One. 2017 Jul 26;12(7):e0181723. doi: 10.1371/journal.pone.0181723 (PMC5528829; doi:10.1371/journal.pone.0181723)
Supplement: S3 Table — ImageJ software was used to count bacterial replicates in Confocal Laser Scanning Micrograph in Fig 5. Four plates were prepared for each type of transformation and confocal micrograph was captured for each plate. (DOCX) [file pone.0181723.s007.docx]

**S3 Table.** Raw Data for number of bacterial replicates transformed with *NS2* gene+Gel.NPs, *NS2* gene alone and Gel.NPs alone. ImageJ software was used to count bacterial replicates in Confocal Laser Scanning Micrograph in Fig 5. Four plates were prepared for each type of transformation and confocal micrograph was captured for each plate.

| Transformation with: | Number of plates (4 plates for each type of Transformation) | Number of transformed bacterial replicates in each confocal micrograph from each plate | Mean  (Average Number of transformed bacterial replicates in 4 confocal micrographs from each type of Transformation) | Standard  Deviation | Standard  Error |
| --- | --- | --- | --- | --- | --- |
| *NS2*gene  +Gel.NPs | 1 | 227 | 236 | 7.63 | 3.82 |
|  | 2 | 245 |  |  |  |
|  | 3 | 233 |  |  |  |
|  | 4 | 238 |  |  |  |
| *NS2* gene alone | 1 | 125 | 119 | 6.06 | 3.03 |
|  | 2 | 118 |  |  |  |
|  | 3 | 122 |  |  |  |
|  | 4 | 111 |  |  |  |
| Gel.NPs alone | 1 | 0 | 0 | 0 | 0 |
|  | 2 | 0 |  |  |  |
|  | 3 | 0 |  |  |  |
|  | 4 | 0 |  |  |  |
