# Supplementary material for: Gelatin nanoparticles enhance delivery of hepatitis C virus recombinant NS2 gene
Source: PLoS One. 2017 Jul 26;12(7):e0181723. doi: 10.1371/journal.pone.0181723 (PMC5528829; doi:10.1371/journal.pone.0181723)
Supplement: S4 Table — ImageJ software was used to count bacterial replicates in confocal micrographs. (DOCX) [file pone.0181723.s008.docx]

**S4 Table.** Number of bacterial replicates transformed with *NS2* gene+Gel.NPs, *NS2* gene alone and Gel.NPs alone. ImageJ software was used to count bacterial replicates in confocal micrographs.

| Transformation with: | **Mean ± SE** |
| --- | --- |
| *NS2*gene+Gel.NPs | 236 **±**3.82*^,a^ |
| *NS2* gene alone | 119 **±**3.03*^,b^ |
| Gel.NPs alone | 0 *^,c^ |

- There is a significant difference between groups by using one way ANOVA at P< 0.05 followed by Duncan multiple comparison test.

-The different letters means that there is a significant difference between groups by using Duncan multiple comparison test (P=0.000).

- *Statistically significant difference compared with other groups using student t- test (P=0.000).
